# Distinct Contributions of the Dorsolateral Prefrontal and Orbitofrontal Cortex during Emotion Regulation

**DOI:** 10.1371/journal.pone.0048107

**Published:** 2012-11-07

**Authors:** Armita Golkar, Tina B. Lonsdorf, Andreas Olsson, Kara M. Lindstrom, Jonathan Berrebi, Peter Fransson, Martin Schalling, Martin Ingvar, Arne Öhman

**Affiliations:** 1 Stockholm Brain Institute, Department of Clinical Neuroscience, Karolinska Institutet, Solna, Sweden; 2 Nordic Center of Excellence in Cognitive Control, Stockholm, Sweden; 3 Institute for Systems Neuroscience, University Hospital Hamburg-Eppendorf, Hamburg, Germany; 4 Mood and Anxiety Program, National Institute of Mental Health, Bethesda, Maryland, United States of America; 5 Center for Molecular Medicine and Surgery, Karolinska Institutet, Solna, Sweden; 6 Osher Center for Integrative Medicine, Stockholm, Sweden; 7 NIH Center for the Study of Emotion and Attention, Gainesville, Florida, United States of America; Federal University of Rio de Janeiro, Brazil

## Abstract

The lateral prefrontal and orbitofrontal cortices have both been implicated in emotion regulation, but their distinct roles in regulation of negative emotion remain poorly understood. To address this issue we enrolled 58 participants in an fMRI study in which participants were instructed to reappraise both negative and neutral stimuli. This design allowed us to separately study activations reflecting cognitive processes associated with reappraisal in general and activations specifically related to reappraisal of negative emotion. Our results confirmed that both the dorsolateral prefrontal cortex (DLPFC) and the lateral orbitofrontal cortex (OFC) contribute to emotion regulation through reappraisal. However, activity in the DLPFC was related to reappraisal independently of whether negative or neutral stimuli were reappraised, whereas the lateral OFC was uniquely related to reappraisal of negative stimuli. We suggest that relative to the lateral OFC, the DLPFC serves a more general role in emotion regulation, perhaps by reflecting the cognitive demand that is inherent to the regulation task.

## Introduction

The capacity to regulate the generation, experience and expression of emotion is a central aspect of mental health [1]. Indeed, emotion dysregulation is a common feature of psychiatric illness, and particularly of depressive and anxiety disorders [2]. The clinical implications of emotional regulation have promoted a growing interest in understanding the neural aspects of emotion regulation. Available data suggest that emotion regulation is accomplished through the interaction between the prefrontal cortex (PFC) – involved in control processes – and subcortical structures, such as the amygdala, involved in the generation of emotions [3].

Instruction-induced reappraisal of an emotionally salient stimulus is the most extensively studied form of cognitive emotion regulation. During reappraisal, cognitive strategies are used to reinterpret the meaning of an emotion-eliciting stimulus in terms of a non-emotional event thereby attenuating automatic emotional responses [1]. Neuroimaging studies of reappraisal collectively suggest that efforts to reappraise an emotional stimulus activate wide areas of the PFC, including ventrolateral, ventromedial, dorsolateral and dorsomedial regions [4]–[10]. The variety of PFC regions implicated in these past studies is likely to reflect important differences in specific experimental manipulations [11]. The basic rationale underlying reappraisal paradigms is that the emotion regulatory processes can be isolated by subtracting the effect of prototypical attend trials, during which subjects passively view negative pictures, from reappraise trials when subjects engage in a cognitively demanding emotional regulation task [12]. However, the direct comparison between attend and reappraise trials does not allow for a differentiation of activations due to emotion regulation *per se* and activations reflecting the recruitment of networks that are shared between different cognitive regulation processes more generally. Although previous research confirms that such a general network of cognitive control [13] are likely to be important in emotion regulation, the question remains open whether there are regions in the brain that that are uniquely related to attempts at regulating negative emotion. In fact, consistent with the notion of a general cognitive control network [13], previous studies of reappraisal commonly report activations of areas that are shared with those reported from studies of the cognitive control of other mental processes, such as memory and attention [14]–[19]. Specifically, the consistent recruitment of DLPFC during reappraisal [11] may reflect a more general role of this area in maintaining and representing the attentional demands of the task [20] and in monitoring and manipulating information held in working memory [20]–[22]. Because of the sparse connections between the DLPFC and the amygdala [23], this region is likely to influence the amygdala indirectly by modulating activity in other regions, such as perceptual areas in the parietal/occipital cortex or the orbitofrontal cortex (OFC), which are directly connected to the amygdala [4], [12]. The lateral OFC is of particular interest, because it is critically involved in cognitive control functions, such as response inhibition and response selection [14], [24], as well as in various forms of cognitive emotion regulation [25]–[27] and in particular reappraisal [5], [7], [8], [28]. Moreover, previous studies on reappraisal of negative affect have reported reappraisal-dependent coupling between the lateral OFC and the amygdala [28], [29] and activity in the right lateral OFC has been identified as a core region mediating successful reappraisal of negative emotion [30].

Although there are other candidate regions with relevance for emotion regulation, such as the subgenual anterior cingulate cortex [29], [31], no study has so far addressed the relative contribution of the DLPFC and lateral OFC during reappraisal. In the present study, we tested the prediction that the DLPFC serves a more general cognitive control function in emotion regulation, whereas the lateral OFC is uniquely engaged in inhibition of negative emotions during an instruction-induced reappraisal task. To address this issue, we used a 2×2 factorial design crossing factors of instruction (reappraisal vs. no reappraisal) and stimulus valence (emotional vs. non-emotional). This design allowed us to separate activations related to general cognitive control processes (main effect of instruction) and those specifically devoted to the control of negative emotion (instruction × stimulus valence interaction), and has recently been used in other emotion regulation tasks such as during distancing [27] and detachment [19] from negative emotion. Thus, we hypothesized that the DLPFC would be activated across conditions requiring reappraisal (general contribution), whereas the OFC would be uniquely activated during conditions involving reappraisal of negative emotion (unique contribution).

## Materials and Methods

### Ethics Statement

The protocol was approved by the ethics committee of Karolinska Institutet.

### Participants

We recruited 61 participants who were free from self-reported life-time psychiatric or neurological disease and medication. Prior to analysis, we excluded 2 left handed participants and 1 participant with abnormal brain anatomy leaving a final sample of 58 (26 men) right-handed, participants with a mean age of 24.02 years (SD = 2.26). All participants gave informed consent and were paid 400 SEK (approximately 55 USD) for their participation. Due to technical problems, the behavioral rating data were missing for 2 participants, who therefore were excluded from the statistical analyses of the rating data.

### Stimuli

Forty negative and 40 neutral pictures were selected based on normative ratings from the International Affective Picture System (IAPS) [32]. Each category of negative and neutral stimuli was divided into two sets (A and B) consisting of 20 negative and 20 neutral stimuli each so that order of presentation and coupling between picture set (A/B) and instruction (Reappraise/Attend) was balanced between participants. Two-tailed *t*-tests yielded significantly lower valence (*p*<.05 ) and higher arousal (*p*<.05 ) ratings for negative compared to neutral pictures, whereas there were no differences in arousal or valence between the two negative picture sets (*p*>.05) or between the two neutral picture sets (*p*>.05) (see Table 1). Stimuli were displayed via fMRI-compatible goggles (NordicNeuroLab, Bergen, Norway) and the experiment was programmed in Presentation® v.14 (Neurobehavioral Systems Inc., Albany, CA).

**Table 1:** Mean valence and arousal ratings for the negative and neutral stimuli.

	Valence	Arousal
**Negative**	1.9 (0.34)	6.5 (0.59)
**Neutral**	4.9 (0.55)	3.01 (0.90)

Standard deviations (SD) are shown in parenthesis.

### Procedure

**Figure 1:**
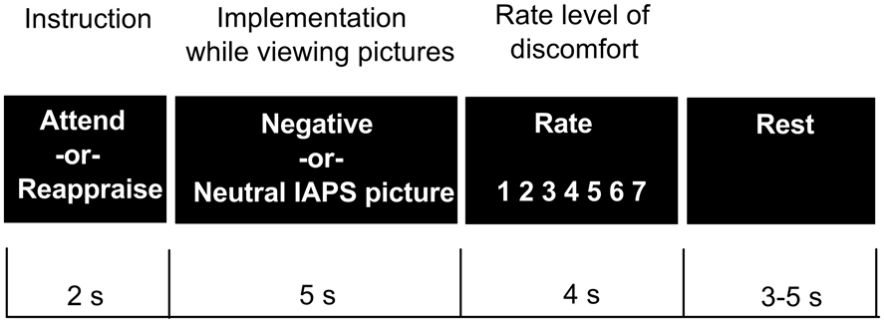
Experimental procedure. Each trial began with an initial 2 sec instruction to either “Attend” or “Reappraise” which was followed by a 5 sec presentation of a negative or a neutral picture. During attend trials, participants were instructed to attend to the picture without trying to alter any ongoing feelings and on reappraise trials they were instructed to neutralize their feelings to the picture. After each picture viewing phase, participants indicated their current level of discomfort on a scale from 1 to 7 (1 = minimal or no discomfort, 7 = maximum discomfort). Each trial ended with a fixation cross jittered between trials with a duration of 3–5 seconds.

Most previous studies on instruction-induced emotion regulation have typically excluded a control condition involving a regulation instruction preceding neutral picture trials. The basic rationale for this was described in an early emotional suppression study [33], in which participants reported confusion when instructed to suppress emotional reactions to neutral pictures. However, two more recent studies have reported successful compliance during regulation of neutral trials, using distancing [27] and detachment [19] as regulation instructions. Therefore, in the current study, we performed extensive pilot testing to confirm that participants could comply with the instructions Attend (i.e. “passively view the picture without trying to manipulate ongoing responses”) and Reappraise (i.e. “relate to the picture in non-emotional terms by reinterpreting the content of the picture so that it no longer elicits a response”) independently of whether they preceded neutral or negative trials. An example of an instruction that was given for Reappraise neutral trials was “even if you do not experience the picture to be very emotional, you should maintain the regulation instruction and reinterpret the meaning of the picture in order to further neutralize your experience of the picture”. Before beginning the experiment, all participants performed 12 practice trials that mirrored the experimental procedure, gave verbal confirmation on understanding how to comply with the instructions, and gave a short description of how they were to do so. No participants reported confusion about how to adopt a reappraisal strategy for the neutral and negative trials before or after completing the experimental task. The experiment consisted of four types of trials: 20 attend negative, 20 reappraise negative, 20 attend neutral and 20 reappraise neutral. Trial presentation was pseudo-randomized with no more than two consecutive trials of the same condition. On each trial, participants were given a 2 s written instruction on the screen (attend/reappraise) after which they viewed a picture (negative/neutral) for 5 s. After each picture viewing phase, subjects rated their current level of discomfort on a scale ranging from 1 to 7, on which 1 = minimal or no discomfort and 7 = maximal discomfort. This was done in order to assess the affective experience of the participant after completing each trial. Each trial ended with the presentation of a fixation cross that was jittered 3–5 s between trials (Fig. 1). Ratings were performed on a MRI-compatible joystick (Mag Design and Engineering Sunnyvale, CA; www.magconcept.com).

### Image Acquisition

An anatomical scan and fMRI data were obtained using a GE Sigma Echo Speed 1.5 T scanner and an 8-channel head-coil. Functional scans were obtained using a gradient echo sequence T2*-weighted echoplanar imaging (EPI) scan (TR = 2.5 sec, TE = 40 ms, flip angle = 90 degrees, 32 axial slices in ascending order (thickness = 3 mm with 1 mm gap) and a field of view (FOV) = 22 cm×22 cm, matrix size = 64×64). The first ten scans were defined as dummy scans to allow for longitudinal T1-equilibrization and these were not included in the analysis. Functional image acquisition was divided into 2 runs of 245 volumes with a break of approximately 20–30 sec between the runs. A T1-weighted structural image (3D-SPGR) was also collected at the end of the experiment.

### Image Preprocessing and Analysis

All imaging data were analyzed with SPM8 (Statistical parametric mapping, The Welcome Department of Imaging Neuroscience, Institute of Neurology, University College London) running on Matlab20010a (MathWorks, Natick, MA). For each subject, individual images were first slice-time corrected to adjust for acquisition time differences between the slices and realigned to the first volume to correct for head movement. The T1-weighted image was then co-registered with the mean EPI image and subsequently segmented using the unified segmentation approach as implemented in the “New Segment” routine in SPM8 to generate gray and white matter images. These images were then used within the DARTEL toolbox to create structural templates across subjects as well as individual flow fields that were then used to spatially normalize the EPI images into MNI space. Finally, all images were spatially smoothed with a Gaussian kernel with a full width at half maximum (FWHM) of 8 mm and temporally high-pass filtered with a cutoff of 128 to remove low-frequency drifts.

Statistical analysis was performed using standard procedures for fMRI involving a fixed effect model at the single-subject level and a random effects model at the group level [34]. For each participant, boxcar regressors representing the 5 s duration of each condition (Reappraise negative, Attend negative, Reappraise neutral, Attend neutral) was convolved with the canonical hemodynamic response function and subsequently entered into a general linear model as implemented in SPM. Realignment parameters were included as regressors of no interest to account for variance related to movement. In a second level random effects group analysis, we specified a 2 (Instruction) × 2 (Valence) within-subject ANOVA with non-sphericity correction using the flexible factorial option in SPM8.

Given the role of the amygdala in emotional processing [35] and that this region has been shown to be modulated by reappraisal [3], [36], we defined the amygdala as an a priori region of interest (ROI). To confirm that our task successfully engaged the amygdala, we looked at the main effect of Valence (Attend negative + Reappraise negative > Attend neutral + Reappraise neutral). The ROI was created around the right and left amygdala using the automated anatomical labels of the Wake Forest University (WFU) PickAtlas toolbox [37]. Activation within the ROI was considered significant at p<.05, family-wise error (FWE) corrected together with a 10 voxel cluster threshold. To investigate activations that were related to the reappraisal instruction in general, we looked at the main effect of Instruction (Reappraise negative + Reappraise neutral > Attend negative + Attend neutral). Finally, to address activations that were specifically related to reappraisal of negative emotion, we looked at the Instruction × Valence interaction given by the interaction term Reappraise (negative- neutral) > Attend (negative – neutral). Given our a priori hypothesis regarding the contributions of the DLPFC and the lateral OFC in emotion regulation, these regions were predefined as ROIs (DLPFC = middle frontal gyrus; lateral OFC = inferior and middle orbital gyri) using the automated anatomical labels of the Wake Forest University (WFU) PickAtlas toolbox [37]. Unless stated otherwise, activation within the ROIs was considered significant at *p*<.05, FWE together with a 10 voxel cluster threshold. To more fully explore the neural regions related to regulation, we supplemented these ROI analyses with whole brain analyses that were considered significant at *p*<.05, FWE corrected, together with a cluster threshold of 10 voxels (see Supplementary data). All reported coordinates refer to Montreal Neurological Institute (MNI) space.

## Results

### Behavioral Ratings

**Figure 2:**
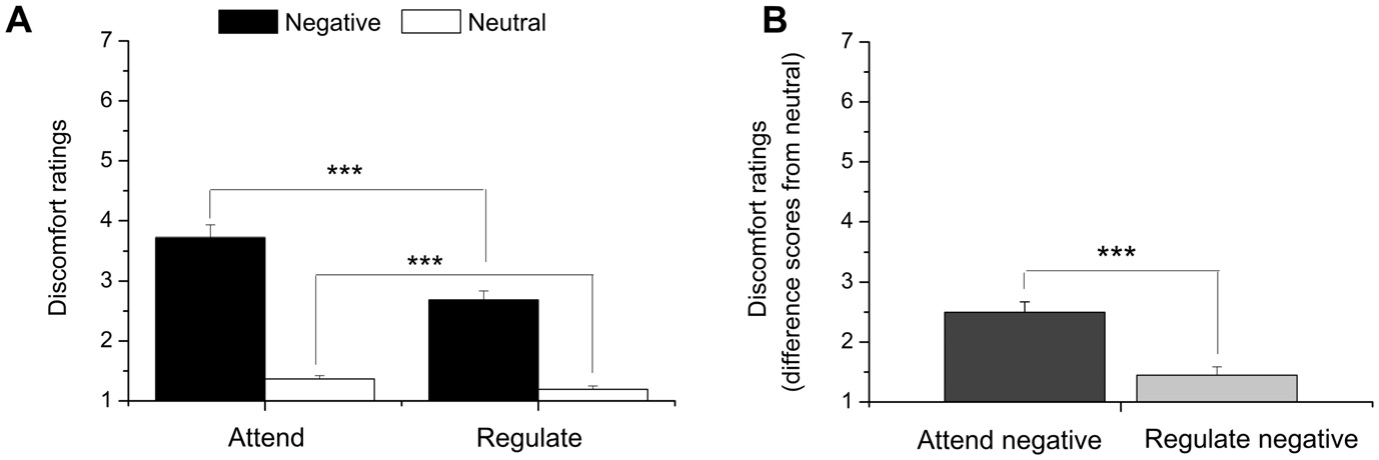
Behavioral results. Discomfort ratings were significantly lower during **A**. Reappraise negative vs. Attend negative trials and during Reappraise neutral vs. Attend neutral trials. **B**. Successful emotion regulation as revealed by significantly lower subjective ratings of discomfort during Reappraise negative trials compared to Attend negative trials expressed as difference scores from neutral trials. Errors bars represent SEM. *** = *p*<.001.

To index whether the images were experienced as less negative after regulation, participants were instructed to rate their current level of discomfort after each picture-viewing phase. As expected, discomfort ratings were lower on Reappraise than on Attend trials both for negative and neutral stimulus pictures (Fig. 2A). A 2 (Instruction: reappraise vs. attend) × 2 (Valence: negative vs. neutral) repeated-measures analysis of variance (ANOVA), resulted in significant main effects of Instruction *F*(1, 55) = 83.55, *p*<.001, η^2^ = .60 and Valence *F*(1, 55) = 194.31, *p*<.001, η^2^ = .78, as well as a significant Instruction × Valence interaction *F*(1, 55) = 60.22, *p*<.001, η^2^ = .52. The reliable interaction was driven by significantly lower discomfort ratings on Reappraise (negative-neutral) than on Attend (negative–neutral) trials. To confirm this interpretation, we first subtracted ratings during the viewing of neutral pictures from ratings during the negative pictures for each instruction and then compared these difference terms, which resulted in a highly significant difference, *p*<.001 (Fig. 2B).

### Amygdala Activation

First, to confirm that our experimental task effectively engaged the amygdala when participants were exposed to negative pictures, we looked at the main effect of Valence within the predefined amygdala ROI. As expected, the analysis revealed a significant bilateral amygdala activation in response to negative compared to neutral pictures (right amygdala: x = 20, y = −2, z = 18, *T* = 12.19, *k*_E_ = 566 voxels; left amygdala: x = −2, y = −3, z = −18, *T = *10.10, *k*_E_ = 508 voxels), *p*<.05, (FWE). More specifically, we wanted to confirm that we (a), accomplished successful emotion elicitation, as demonstrated by greater amygdala activation in the contrast Attend negative > Attend neutral [4], [38], and (b), that reappraisal had a down-regulating effect on the amygdala response, as demonstrated by a greater amygdala activity in the contrast Attend negative > Reappraise negative [11]. One-sample t-tests confirmed a significantly larger bilateral amygdala activation in the Attend negative > Attend neutral condition (right amygdala x = 21, y = 0, z = −19, *T = *4.24, *k*_E_ = 192 voxels; left amygdala x = −20, y = 1, z = −18, *T = *4.80, *k*_E_ = 192 voxels) as well as during the Attend negative > Reappraise negative condition (right amygdala x = 20, y = −2, z = −18, *T = *11.13, *k*_E_ = 564 voxels; left amygdala x = −18, y = −2, z = −16, *T = *9.25, *k*_E_ = 508 voxels), *p*<.05, (FWE).

### Main Effect of Instruction

**Figure 3:**
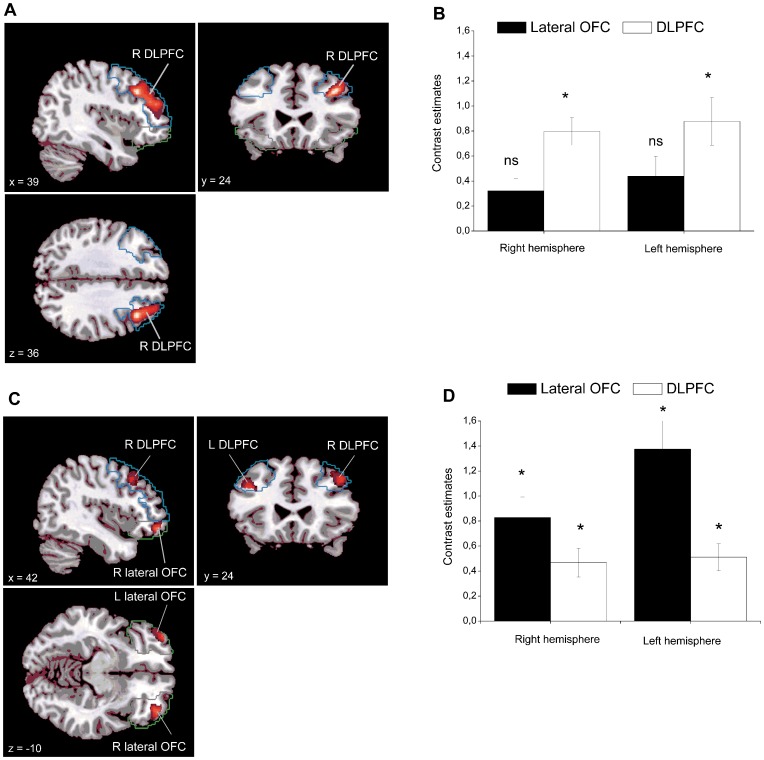
Statistical parametric maps showing activations during the main effect of instruction and the Instruction× Valence interaction (A). Activation in the right dorsolateral prefrontal cortex (DLPFC) during the main effect of instruction across stimulus valence in Reappraise (negative + neutral) > Attend (negative + neutral). Significant activations are displayed overlaid on the DLPFC region-of-interest (ROI) image (shown in blue) in sagittal (upper left), coronal (upper right) and axial (lower left) view, respectively and the (**B**) corresponding contrast estimates for the right and left DLPFC and lateral OFC at peak activation coordinates [right doroslateral PFC: x = 39, y = 24, z = 36; left DLPFC: right OFC; x = 26, y = 46, z = −13; left OFC: x = −36, y = 46, z = −12]. Note that the activations were not significant in the lateral OFC. (**C**) Activation in the right orbitofrontal cortex (OFC) and the right DLPFC during the Instruction × Valence interaction contrast Reappraise (negative - neutral) > Attend (negative - neutral). Significant activations are displayed overlaid on the DLPFC ROI (shown in blue) and the lateral OFC ROI (shown in green) in sagittal (upper left panel), coronal (upper right panel) and axial (lower left panel) view, and the (**D**) corresponding contrast estimates during the main effect of instruction and the Instruction × Valence interaction contrasts for the right and left DLPFC and lateral OFC at peak activation coordinates [right DLPFC: x = 46, y = 21, z = 44; left DLPFC; x = −39, y = 24, z = 32; right OFC; x = 42, y = 50, z = −10; left OFC: x = −38, y = 60, z = −4]. All activations were thresholded at *p*<.05 (FWE) and were based on the DLPFC and lateral OFC ROIs. * = *p*<.05 (FWE). Error bars represent standard error of the mean.

To test our hypothesis that activation in the DLPFC would be significantly related to the reappraisal instruction across picture valence, we first investigated the effect of the reappraisal instruction. In line with our hypothesis, the instruction to “Reappraise” compared to “Attend” resulted in significant activations only in the DLPFC ROI (Fig. 3A and 3B and Table 2) but not in the lateral OFC ROI. A whole brain analysis revealed significant activation in a fronto-parietal attentional control network involving dominantly right-sided activations in DLPFC and inferior parietal lobe (see Table S1).

**Table 2:** Group activations during Reappraise > Attend collapsed across Stimulus valence (negative/neutral) and during interaction between Instruction (Reappraise vs. Attend) and Stimulus Valence (negative vs. neutral).

	Peak coordinates						
Region	BA	Side	Numberofvoxels	x	y	z	*T*
**Main effect of Instruction:**Reappraise (negative + neutral) > Attend (negative + neutral)							
**Middle frontal**	**BA9**	**R**	**3557**	39	24	36	6.97
		**L**	**20**	−38	48	14	4.50
**Instruction** × **Stimulus valence interaction:**Reappraise (negative − neutral) > Attend (negative − neutral)							
**Orbitofrontal**	**BA10**	**R**	**98**	42	50	−10	5.04
		**L**	**217**	−38	60	−4	5.68
**Middle Frontal**	**BA9**	**R**	**34**	46	21	44	4.61
		**L**	**56**	−39	24	32	4.76

All reported activations are based on anatomical ROI analysis and significant at *p*<.05 (FWE).

Note: BA = Brodmann area; R = Right; L = Left. Coordinates: MNI system.

### Instruction × Valence Interaction

To investigate the brain areas devoted to reappraisal of negative emotion when controlling for the main effect of instruction, we tested the effect of the Instruction × Valence interaction within the DLPFC and the lateral OFC ROIs This contrast revealed mainly bilateral activation in the lateral OFC and to smaller extent activation within the DLPFC (Fig. 3C and 3D and Table 2) A whole brain analysis did not reveal any additional prefrontal areas (see Table S2).

### Simple Effects of Regulation

**Figure 4:**
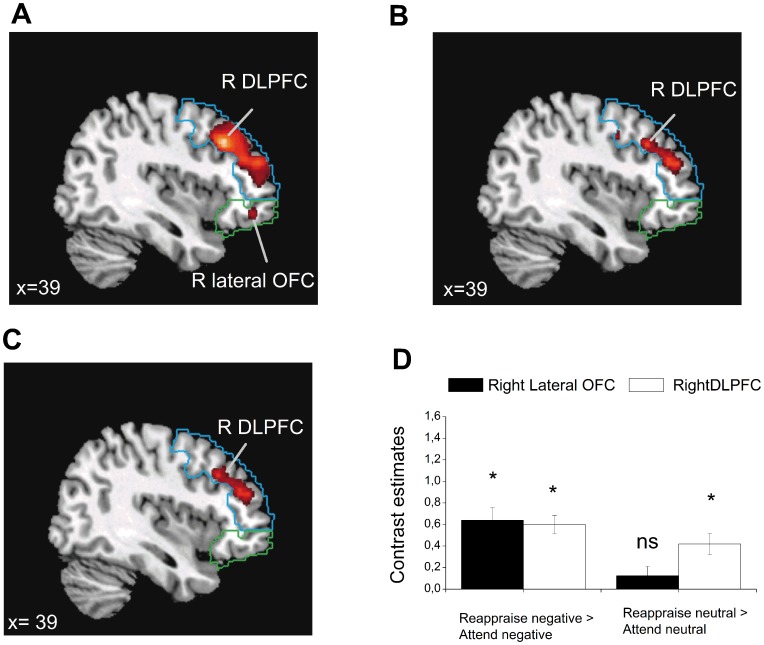
Statistical parametric maps showing activations during reappraisal of negative and neutral stimuli. (**A**) Activation in the right dorsolateral prefrontal cortex (DLPFC) and the lateral orbitofrontal cortex (OFC) in the Reappraise negative > Attend negative and (**B**) in the right DLPFC during Reappraise neutral > Attend neutral, and (**C**) overlapping activations in the right DLPFC in the conjunction analysis of the common effect of Reappraise negative > Attend negative and Reappraise neutral > Attend neutral. Significant activations are displayed overlaid on the DLPFC region-of-interest (ROI) image (shown in blue) and the lateral OFC ROI (shown in green) in sagittal view. (**D**) Contrast estimates extracted for the right DLPFC and the right lateral OFC during the Reappraise negative > Attend negative contrast [DLPFC: x = 42, y = 24, z = 39; lateral OFC: x = 42, y = 45, z = −9] and the Reappraise neutral > Attend neutral contrast [right DLPFC: x = 33, y = 43, z = 30; right lateral OFC x = 25, y = 36, z = −8]. Note that the activations were not significant for the lateral OFC in Reappraise neutral > Attend neutral or in the conjunction analysis. Activations were thresholded at *p*<.001 (uncorrected) for illustrative purposes and were based on the DLPFC and lateral OFC ROIs. * = *p*<.05 (FWE). * = *p*<.05 (FWE). Error bars represent standard error of the mean.

To further investigate the Instruction × Valence interaction and in parallel to the results reported for the behavior data, we additionally ran one-sample t-tests comparing activations in Reappraise vs. Attend to neutral and negative trials separately within the DLPFC and lateral OFC ROIS. As predicted, whereas the DLPFC was significantly activated in both reappraisal contrasts irrespectively of whether reappraisal effort was targeted on negative (Reappraise negative > Attend negative) or neutral stimuli (Reappraise neutral > Attend neutral), the lateral OFC was uniquely activated during reappraisal of negative stimuli (Fig. 4 and Table 3). To confirm that the specificity of these effect could not simply be ascribed to differences in degree of activation or due to thresholding, we additionally tested the effect of Reappraise neutral > Attend neutral within the lateral OFC ROI at a more liberal threshold of *p<*.01 uncorrected. This analysis still did not reveal any activation within the lateral OFC during the neutral condition (see Table S3 for results from the whole brain analysis).

**Table 3:** Group activations for “Reappraise” vs. “Attend” during negative and neutral trials.

	Peak coordinates						
Region	BA	Side	Number of voxels	x	y	z	*T*
**Reappraise negative > Attend negative**							
**Middle frontal**	**BA9**	**R**	**3855**	42	24	39	7.11
**Orbitofrontal**	**BA10**	**R**	**195**	42	54	−9	5.32
		**L**	**572**	−34	54	−3	5.72
**Reappraise neutral > Attend neutral**							
**Middle frontal**	**BA9**	**R**	**131**	26	46	18	5.68
**Conjunction analysis:**Reappraise negative > Attend negative and Reappraise neutral > Attend neutral							
**Middle frontal**	**BA9**	**R**	**338**	27	49	20	5.21

All reported activations are based on anatomical ROI analysis and significant at *p*<.05 (FWE).

Note: BA = Brodmann area; R = Right; L = Left. Coordinates: MNI system.

For clarity, we also confirmed that neither of our prefrontal ROIs was significantly more activated during reappraisal of neutral trials as compared to during the reappraisal of negative trials. Neither ROI showed significant activation in Reappraise neutral > Reappraise negative (*p*<.05, FWE).

### Common Effects of Regulation

Finally, we wanted to establish that activation of the DLPFC was shared between the reappraisal contrasts and that this shared activation was specific to the right DLPFC. Therefore, we conducted a conjunction analysis testing overlapping activity during the contrast Reappraise negative > Attend negative and Reappraise neutral > Attend neutral within the right DLPFC. As predicted, the conjunction analysis revealed significant overlapping activity in the right DLPFC (Fig. 4C and Table 3), confirming that the activation in DLPFC was not restricted to reappraisal of negative stimuli. Testing for an effect within the lateral OFC ROI at a more liberal threshold of *p<.*01 uncorrected still did not reveal activation in the lateral OFC that was shared between the negative and neutral regulation contrasts. Results from the whole brain analysis did not reveal significant activations in any prefrontal regions apart from the DLPFC. In addition, this analysis revealed activity in a region in the inferior parietal cortex (see Table S4).

## Discussion

In line with our hypothesis regarding the distinct roles of the DLPFC and the lateral OFC in emotion regulation, our results showed that the DLPFC was activated across the reappraisal task irrespectively of whether the reappraisal effort was targeted on negative or neutral stimuli. In contrast, the lateral OFC was uniquely engaged during reappraisal of negative stimuli.

First, our task resulted in larger amygdala responses to negative as compared to neutral stimuli, indicating that negative stimuli elicited a greater emotional response. Second, we demonstrated that reappraisal instructions resulted in down-regulation of the larger amygdala response to negative stimuli. In terms of brain regions activated during reappraisal, we found a main effect of the reappraisal task across stimulus valence that was restricted to the DLPFC and the inferior parietal cortex (see Table S1). By demonstrating robust activation in the fronto-parietal attentional control network, we showed that the effect of the regulation task across both negative and neutral stimulus overlapped with the effect commonly reported for the main emotion regulation contrast (i.e. Reappraise negative > Attend negative) in previous studies [4], [6]–[8], [19], [39]. Furthermore, the DLPFC was not uniquely related to reappraisal of negative emotion. Rather, the DLPFC was the only prefrontal region that was significantly activated during reappraisal of both negative and neutral stimuli. Critically, we identified voxels within the DLPFC that showed overlapping activity in both reappraisal conditions, indicating a general activation of this region during implementation of regulation. In light of the observed pattern of activity, we suggest that activity in the DLPFC reflects the effortful processing that accompanies reappraisal, perhaps by maintaining the attentional demands of the task. Although different cognitive processes may give rise to the same pattern of brain activation, our interpretation is consistent with previous research using other cognitive control tasks to demonstrate that DLPFC activity increases with increasing task load [40], and that activity within this region represents the active maintenance of task-related requirements for attentional control [20].

Assuming that activity in the DLPFC reflects cognitive processing that is inherent to the reappraisal task, it is therefore not surprising that activity within this region was observed across negative and neutral conditions involving the reappraisal instruction. Consequently, this gave rise to activation patterns that were shared with the lateral OFC, as was seen in activation in both regions during reappraisal of negative emotion. Importantly though, unlike the general recruitment of the DLPFC across reappraisal conditions, activity in the lateral OFC was uniquely observed during reappraisal of negative stimuli. The location and functional neuroanatomy of the OFC makes it ideally suited for suppressing neural activity in subcortical structures due to its rich reciprocal connectivity to both the PFC and emotion generating processing regions [23]. A comprehensive meta-analysis on the role of the OFC has suggested that subregions within this region serve functionally distinct roles [41]. According to this view, while activity in the medial region of the OFC is related to monitoring, learning and memory of the reward value of reinforcing stimuli, activity within the lateral regions of the OFC is more related to evaluation of punishers that can promote behavioral change [42]. In line with a medial-lateral division within the OFC, previous reappraisal studies have demonstrated an association between increased medial OFC activity and reactivity to emotional material [4], [7], whereas lateral OFC activity has been associated with down-regulation of emotional responses [5], [7], [27], and a functional coupling with the amygdala during reappraisal [28], [29]. In fact, in a mediation analysis reported by Wager and colleagues [30], the lateral OFC was identified as a core region mediating reappraisal success through two independent subcortical pathways involving the nucleus accumbens that predicted successful reappraisal, and the amygdala, that predicted unsuccessful reappraisal. These results were interpreted to suggest that the lateral OFC is engaged both by a negative appraisal process that involves the amygdala, and a positive appraisal process involving the nucleus accumbens. As such, our results are supported by these data as they implicate the lateral OFC as critically involved in the regulation of emotion. Moreover, our results fits particularly well with a recent study [28] that directly contrasted emotion regulation accomplished by two different regulation strategies and reported that the lateral OFC was specifically engaged by reappraisal as compared to when using the strategy of distancing. Importantly, the present results, which directly compared the relative contribution of the DLPFC and the lateral OFC during reappraisal, are consistent with the proposed role of these regions in other regulation tasks, such as during the regulation of pain [20], [43]–[45] and selective attention [13], [46].

Although our findings point to a specific contribution of the lateral OFC during down-regulation of negative emotion, previous studies have also implicated this region during regulation of positive emotion [8] and during up-regulation of negative emotion (but see [5], [38] for opposite findings). Nevertheless, directly contrasting specific aspects of emotion regulation, such as contrasting different regulation strategies [28], down- and up-regulation of emotion [5], [9], [38], and regulation effort targeted on positive and negative emotion [8], represent related strategies to address the specific contributions of different prefrontal regions during emotion regulation.

An important limitation of the present findings lies in the nature of the reappraisal task. Although widely used as model of instructed emotion regulation [11], the task suffers from limited experimental control as there are no means by which it is possible to confirm exactly what is going on during the different instructions, or exactly what strategies are being employed at a given point in time. This matter is of particular relevance for the interpretation of the current findings, which rest on the assumption that participants could comply with the reappraisal instruction during both negative and neutral trials. As is the case with previous reappraisal studies [3] successful compliance with the instructions can only be obtained by self-reports and subjective ratings after each trial, which are likely to be biased by experimental demands. In the present study, and in previous studies adopting a balanced design by crossing factors of Instruction (regulate vs. passive viewing) with Stimulus valence (negative vs. neutral) [19], [27], participants were asked to rate their response not only to negative, but also to neutral stimulus pictures, which might have introduced further complications [33]. To make sure that our participants understood the rating procedure, they were exposed to an extensive training session, in which they were encouraged to ask questions and to discuss their task until they felt confident that they knew how to complete it successfully. As a result, they did not report any confusion about how to adopt a reappraisal strategy during negative and neutral trials. The self-reported data however still allow for alternative interpretations. Although participants are likely to have complied with the instruction to reappraise on neutral trials (i.e. they experienced stimuli as more neutral after reappraisal), the drop in discomfort ratings could reflect that participants were merely complying with the demands imposed by the experimental setup (i.e. reporting something that believed to comply with the instruction). In future studies, it might be informative to include ratings of subjective experience of effort, which presumably are less susceptible to experimental demands and that can be more directly related to activity in prefrontal areas such as the DLPFC that, at least partly, may reflect task demand.

Taken together, this study is the first to directly contrast the relative contribution of two key regions during reappraisal; the DLPFC and the lateral OFC. We argue that (a) the DLPFC and the lateral OFC both contribute to negative emotion regulation, but (b) that these regions partly reflect different aspects of the emotion regulation process. Critically, our results showed that in contrast to the more general pattern of activation in the DLPFC, the lateral OFC was specifically and uniquely activated during reappraisal of negatively valenced stimuli. These findings add to a growing literature focused on disentangling the contributions of different prefrontal regions during emotion regulation.

## Supporting Information

Table S1 **Whole-brain activations for the main effect of Instruction.** BA = Brodmann area; R = Right; L = Left. Coordinates: MNI system. All reported activations are significant at *p*<.05 (FWE).
(DOC)

Table S2 **Whole-brain activations for the effect of Instruction** × **Valence interaction.** BA = Brodmann area; R = Right; L = Left. Coordinates: MNI system. All reported activations are significant at *p*<.05 (FWE).
(DOC)

Table S3 **Whole-brain activations for “Reappraise” vs. “Attend” during negative and neutral trials.** BA = Brodmann area; R = Right; L = Left. Coordinates: MNI system. All reported activations are significant at *p*<.05 (FWE).
(DOC)

Table S4 **Whole brain activations in the conjunction analysis of Reappraise negative > Attend negative and Reappraise neutral > Attend neutral.** BA = Brodmann area; R = Right; L = Left. Coordinates: MNI system. All reported activations are significant at *p*<.05 (FWE).
(DOC)
