# Supplementary material for: Distinct Contributions of the Dorsolateral Prefrontal and Orbitofrontal Cortex during Emotion Regulation
Source: PLoS One. 2012 Nov 7;7(11):e48107. doi: 10.1371/journal.pone.0048107 (PMC3492343; doi:10.1371/journal.pone.0048107)
Supplement: Table S1 — Whole-brain activations for the main effect of Instruction. BA = Brodmann area; R = Right; L = Left. Coordinates: MNI system. All reported activations are significant at p<.05 (FWE). (DOC) [file pone.0048107.s001.doc]

**Table S1**

|  | **Peak coordinates__** | | | | | | | | |
| --- | --- | --- | --- | --- | --- | --- | --- | --- | --- |
| **Region** | | **BA** | **Side** | **Nr of**  **voxels** | **x** | **y** | **z** | ***T*** |  |
| **Activations by Reappraise (negative + neutral) > Attend (negative + neutral)** | | | | | | | | | |
| **Inferior parietal** | | BA40 | R | 3219 |  |  |  |  |  |
|  | |  |  |  | 57 | -41 | 39 | 9.91 |  |
|  | |  |  |  | 51 | -47 | 53 | 6.58 |  |
|  | |  | L | 950 |  |  |  |  |  |
|  | |  |  |  | -58 | -51 | 45 | 7.08 |  |
|  | |  |  |  | -64 | -51 | 33 | 5.60 |  |
| **Middle frontal** | | BA9 | R | 3050 |  |  |  |  |  |
|  | |  |  |  | 39 | 34 | 36 | 6.97 |  |
|  | |  |  |  | 33 | 27 | 32 | 6.92 |  |
|  | |  |  |  | 27 | 51 | 20 | 6.82 |  |
| **Superior frontal** | | BA6/8 | R | 62 |  |  |  |  |  |
|  | |  |  |  | 20 | 13 | 60 | 5.24 |  |

|  |
| --- |
