# Supplementary material for: Distinct Contributions of the Dorsolateral Prefrontal and Orbitofrontal Cortex during Emotion Regulation
Source: PLoS One. 2012 Nov 7;7(11):e48107. doi: 10.1371/journal.pone.0048107 (PMC3492343; doi:10.1371/journal.pone.0048107)
Supplement: Table S2 — Whole-brain activations for the effect of Instruction × Valence interaction. BA = Brodmann area; R = Right; L = Left. Coordinates: MNI system. All reported activations are significant at p<.05 (FWE). (DOC) [file pone.0048107.s002.doc]

**Table S2**

|  | **Peak coordinates__** | | | | | | | |  | |
| --- | --- | --- | --- | --- | --- | --- | --- | --- | --- | --- |
| **Region** | **BA** | **Side** | **Nr of voxels** | **x** | **y** | **z** | ***T*** |  | | |
|  |  |  |  |  |  |  |  |  | | |
|  | | | | | | | |  | | |
| **Activation by Reappraise (negative –neutral) > Attend (negative-neutral)** | | | | | | | |  | | |
| **Orbitofrontal** | BA10 | R | 68 |  |  | | | | | |
|  |  |  |  |  |  | | | | | |
|  |  |  |  | 42 | 50 | -10 | 5.04 |  | |  |
|  |  | | | | | | | | | |
|  |  | L | 110 |  |  |  |  |  | |  |
|  |  |  |  | -38 | 60 | -4 | 5.68 |  | |  |
| **Superior**  **frontal** | BA6/8 | L | 28 | -12 | 21 | 54 | 5.55 |  | | |
| **Inferior**  **parietal** | BA40 | R | 120 |  |  |  |  |  | |  |
|  |  |  |  | 50 | -54 | 44 | 5.28 |  | |  |
|  |  |  |  | 46 | -62 | 45 | 5.11 |  | |  |

|  |
| --- |
