# Supplementary material for: Distinct Contributions of the Dorsolateral Prefrontal and Orbitofrontal Cortex during Emotion Regulation
Source: PLoS One. 2012 Nov 7;7(11):e48107. doi: 10.1371/journal.pone.0048107 (PMC3492343; doi:10.1371/journal.pone.0048107)
Supplement: Table S3 — Whole-brain activations for “Reappraise” vs. “Attend” during negative and neutral trials. BA = Brodmann area; R = Right; L = Left. Coordinates: MNI system. All reported activations are significant at p<.05 (FWE). (DOC) [file pone.0048107.s003.doc]

**Table S3**

|  | **Peak coordinates__** | | | | | | | | | | | | | | | | | | | | | | | | | | | | |
| --- | --- | --- | --- | --- | --- | --- | --- | --- | --- | --- | --- | --- | --- | --- | --- | --- | --- | --- | --- | --- | --- | --- | --- | --- | --- | --- | --- | --- | --- |
| **Region** | | **BA** | | | **Side** | | | **Nr of voxels** | | | **x** | | | **y** | | | | **z** | | | | ***T*** | | | |  | | |  |
| **Activations by Reappraise negative > Attend negative** | | | | | | | | | | | | | | | | | | | | | | |  | | | | | | |
| **Inferior**  **parietal** | | BA40 | | R | | | 3281 | | |  | | |  | | | |  | | | |  | | | |  | | | |  |
|  | |  | |  | | |  | | | 56 | | | -47 | | | | 33 | | | | 8.93 | | | |  | | | |  |
|  | |  | |  | | |  | | | 54 | | | -44 | | | | 41 | | | | 8.89 | | | |  | | | |  |
|  | |  | | L | | | 1531 | | |  | | |  | | | |  | | | |  | | | |  | | | |  |
|  | |  | |  | | |  | | | -57 | | | -50 | | | | 45 | | | | 7.81 | | | |  | | | |  |
|  | |  | |  | | |  | | | -54 | | | -63 | | | | 41 | | | | 6.01 | | | |  | | | |  |
|  | |  | |  | | |  | | | -64 | | | -53 | | | | 32 | | | | 5.67 | | | |  | | | |  |
| **Middle**  **frontal** | | BA9 | | R | | | 3072 | | |  | | |  | | | |  | | | |  | | | |  | | | |  |
|  | |  | |  | | |  | | | 42 | | | 24 | | | | 39 | | | | 7.11 | | | |  | | | |  |
|  | |  | |  | | |  | | | 28 | | | 55 | | | | 23 | | | | 6.84 | | | |  | | | |  |
|  | |  | |  | | |  | | | 34 | | | 30 | | | | 33 | | | | 6.50 | | | |  | | | |  |
|  | |  | | L | | | 164 | | |  | | |  | | | |  | | | |  | | | |  | | | |  |
|  | |  | |  | | |  | | | -34 | | | 54 | | | | -3 | | | | 5.72 | | | |  | | | |  |
| **Superior frontal** | | BA6/8 | | R | | | 71 | | |  | | |  | | | |  | | | |  | | | |  | | | |  |
|  | |  | |  | | |  | | | 16 | | | 21 | | | | 59 | | | | 5.13 | | | |  | | | |  |
| **Orbitofrontal** | | BA10 | | R | | | 44 | | | 42 | | | 45 | | | | -9 | | | | 5.32 | | | |  | | | |  |
|  | |  | |  | | |  | | |  | | |  | | | |  | | | |  | | | |  | | | |  |
|  | |  | |  | | |  | | |  | | |  | | | |  | | | |  | | | |  | | | |  |
| **Activations by Reappraise neutral > Attend neutral** | | | | | | | | | | | | | | | | | | | | | | | | | | | | |  |
| **Inferior parietal** | | BA40 | | R | | | 1018 | | | 62 | | | -38 | | | | 39 | | | | 7.32 | | | |  | | | |  |
| **Middle frontal** | | BA9 | | R | | | 123 | | | 24 | | | 46 | | | | 20 | | | | 5.77 | | | |  | | | |  |
| **Insula** | | BA13 | | R | | | 72 | | | 36 | | | 16 | | | | 8 | | | | 5.68 | | | |  | | | |  |
| _________________________________________________________________________ | | | | | | | | | | | | | | | | | | | | | | | | | | | | |  |
|  | | | | | | | | | | | | | | | | | | | | | | | | |  | | | |  |
|  | |  | |  | | |  | | |  | | |  | | | |  | | | |  | | | |  | | | |  |
|  | | | | | | | | | | | | | | | | | | | | | | | | | | | | |  |
|  | |  | |  | | |  | | |  | | |  | | | |  | | | |  | | | |  | | | |  |
|  | |  | |  | | |  | | |  | | |  | | | |  | | | |  | | | |  | | | |  |
|  | |  | |  | | |  | | |  | | |  | | | |  | | | |  | | | |  | | | |  |
|  | |  | |  | | |  | | |  | | |  | | | |  | | | |  | | | |  | | | |  |
|  | |  | |  | | |  | | |  | | |  | | | |  | | | |  | | | |  | | | |  |
|  | |  | |  | | |  | | |  | | |  | | | |  | | | |  | | | |  | | | |  |
|  | |  | |  | | |  | | |  | | |  | | | |  | | | |  | | | |  | | | |  |
|  | |  | |  | | |  | | |  | | |  | | | |  | | | |  | | | |  | | | |  |
|  | |  | |  | | |  | | |  | | |  | | | |  | | | |  | | | |  | | | |  |
|  | |  | |  | | |  | | |  | | |  | | | |  | | | |  | | | |  | | | |  |
|  | |  | |  | | |  | | |  | | |  | | | |  | | | |  | | | |  | | | |  |
|  | |  | |  | | |  | | |  | | |  | | | |  | | | |  | | | |  | | | |  |
|  | |  | |  | | |  | | |  | | |  | | | |  | | | |  | | | |  | | | |  |
|  | |  | |  | | |  | | |  | | |  | | | |  | | | |  | | | |  | | | |  |
|  | |  | |  | | |  | | |  | | |  | | | |  | | | |  | | | |  | | | |  |
|  | |  | |  | | |  | | |  | | |  | | | |  | | | |  | | | |  | | | |  |
|  | |  | |  | | |  | | |  | | |  | | | |  | | | |  | | | |  | | | |  |
|  | |  | |  | | |  | | |  | | |  | | | |  | | | |  | | | |  | | | |  |
|  | | |  | | |  | | |  | | |  | | | |  | | | |  | | | |  | | | |  | |
|  | | |  | | |  | | |  | | |  | | | |  | | | |  | | | |  | | | |  | |
|  | | |  | | |  | | |  | | |  | | | |  | | | |  | | | |  | | | |  | |
|  | | |  | | |  | | |  | | |  | | | |  | | | |  | | | |  | | | |  | |
|  | | | | | | | | | | | | | | | | | | | | | | | | | | | | | |
|  | | | | | | | | | | | | | | | | | | | | | | | | | | | | | |
|  | | |  | | |  | | |  | | |  | | | |  | | | |  | | | |  | | | |  | |
|  | | |  | | |  | | |  | | |  | | |  | | | |  | | | |  | | | |  | |  |
|  | | |  | | |  | | |  | | |  | | |  | | | |  | | | |  | | | |  | |  |
|  | | |  | | |  | | |  | | |  | | |  | | | |  | | | |  | | | |  | |  |
|  | | |  | | |  | | |  | | |  | | |  | | | |  | | | |  | | | |  | |  |
|  | | | | | | | | | | | | | | | | | | | | | | | | | | | | | |
