# Supplementary material for: Distinct Contributions of the Dorsolateral Prefrontal and Orbitofrontal Cortex during Emotion Regulation
Source: PLoS One. 2012 Nov 7;7(11):e48107. doi: 10.1371/journal.pone.0048107 (PMC3492343; doi:10.1371/journal.pone.0048107)
Supplement: Table S4 — Whole brain activations in the conjunction analysis of Reappraise negative > Attend negative and Reappraise neutral > Attend neutral. BA = Brodmann area; R = Right; L = Left. Coordinates: MNI system. All reported activations are significant at p<.05 (FWE). (DOC) [file pone.0048107.s004.doc]

| **Table S4** | | | | | | | | |
| --- | --- | --- | --- | --- | --- | --- | --- | --- |
|  | | | | | | | |  |
| **Peak coordinates** | | | | | | | |  |
| **Region** | **BA** | **Side** | **Nr of voxels** | **x** | **y** | **z** | ***T*** |  |
| __________________________________________________________________________ | | | | | | | | |
|  |  |  |  |  |  |  |  |  |
| **Inferior parietal** | BA40 | R | 851 | 60 | -39 | 39 | 7.32 |  |
|  |  |  |  |  |  |  |  |  |
| **Middle frontal** | BA9 | R | 36 |  |  |  |  |  |
|  |  |  |  | 26 | 49 | 18 | 5.33 |  |
|  |  |  |  |  |  |  |  |  |
| __________________________________________________________________________ | | | | | | | | |
|  | | | | | | | | |
